# The associations between employees’ risky drinking and sociodemographics, and implications for intervention needs

**DOI:** 10.1186/s12889-018-5660-x

**Published:** 2018-06-14

**Authors:** Mikkel Magnus Thørrisen, Jens Christoffer Skogen, Randi Wågø Aas

**Affiliations:** 1 Faculty of Health Sciences, Department of Occupational Therapy, Prosthetics and Orthotics, OsloMet - Oslo Metropolitan University, St. Olavs plass, NO-0130 Oslo, Norway; 2 Department of Health Promotion, Norwegian Institute of Public Health, Bergen, Norway; 3 Center for Alcohol & Drug Research Stavanger University Hospital, Stavanger, Norway; 4 Faculty of Health Sciences, University of Stavanger, Stavanger, Norway; 5 Presenter – Making Sense of Science, Stavanger, Norway

**Keywords:** Alcohol consumption, Risky drinking, Employees, Workplace, Workforce

## Abstract

**Background:**

Harmful alcohol consumption is a major risk factor for ill-health on an individual level, a global public health challenge, and associated with workplace productivity loss. This study aimed to explore the proportion of risky drinkers in a sample of employees, investigate sociodemographic associations with risky drinking, and examine implications for intervention needs, according to recommendations from the World Health Organization (WHO).

**Methods:**

In a cross-sectional design, sociodemographic data were collected from Norwegian employees in 14 companies (*n* = 3571) across sectors and branches. Risky drinking was measured with the Alcohol Use Disorders Identification Test (AUDIT). The threshold for risky drinking was set at ≥8 scores on the AUDIT. Based on WHO guidelines, risky drinkers were divided into three risk categories (moderate risk: scores 8–15, high risk: scores 16–19, and dependence likely risk: scores 20–40). The association between sociodemographic variables and risky drinking were explored with chi square tests for independence and adjusted logistic regression. The risk groups were then examined according to the WHO intervention recommendations.

**Results:**

11.0% of the total sample reported risky drinking. Risky drinking was associated with male gender (OR = 2.97, *p* < .001), younger age (OR = 1.03, *p* < .001), low education (OR = 1.17, *p* < .05), being unmarried (OR = 1.38, *p* < .05) and not having children (OR = 1.62, *p* < .05). Risky drinking was most common among males without children (33.5%), males living alone (31.4%) and males aged ≤39 (26.5%). 94.6% of risky drinkers scored within the lowest risk category. Based on WHO guidelines, approximately one out of ten employees need simple advice, targeting risky drinking. In high-risk groups, one out of three employees need interventions.

**Conclusions:**

A considerable amount of employees (one to three out of ten), particularly young, unmarried males without children and higher education, may be characterised as risky drinkers. This group may benefit from low-cost interventions, based on recommendations from the WHO guidelines.

## Background

Harmful alcohol consumption is a major risk factor for disease, disability and mortality, and has been identified as a causal agent in more than 200 disease and injury conditions [1]. According to the World Health Organization (WHO), harmful alcohol consumption is related to approximately 3.3 million annual deaths globally (5.9% of all mortality worldwide) [2]. Consumption levels have been found to be highest in the developed world.

Alcohol is by far the most used and misused psychoactive substance in the workforce and employees’ alcohol consumption is associated with productivity loss, and therefore with considerable economic costs at a societal level [3]. A recently published systematic review reported that employees’ alcohol consumption is associated with both short- and long-term sickness absence [4]. Some studies also indicate that alcohol consumption is related to sickness presenteeism, i.e., reduced on-the-job productivity [5–7].

Risky drinking may be defined as a drinking pattern that increases the risk of social, legal, medical, occupational, domestic, and economic problems [8]. It is, however, difficult to determine an appropriate cut off for risky drinking, even when assuming a linear relationship between alcohol consumption and harm. What constitutes risky drinking is inextricably linked to individual characteristics. General health, physiological factors, sociodemographic variables as well as lifestyle factors may affect how much a person can drink before adverse consequences emerge [9]. Whereas some definitions of risky drinking are based solely on alcohol consumption (frequency and/or intensity), measured in terms of consumed alcohol units within a specified time frame, other conceptualisations are based on instruments assuming a more complex relationship between alcohol and health [10], such as the Alcohol Use Disorders Identification Test (AUDIT) [8], which defines risky drinking as a sum score equal to or higher than a predefined scale threshold, based on items comprising symptoms of alcohol dependence and alcohol-related problems as well as alcohol consumption.

Risky drinking has been studied within different populations across countries, with prevalence estimates varying between 5.4 and 52.0% [11–16]. In a Norwegian general population sample, it was found that 17.0% of respondents scored within the range of risky drinking [17]. In a national sample of Norwegian students, 46.1% scored above the threshold of risky drinking [18]. Some studies have explored the prevalence of risky drinking within working populations, e.g., among Australian industrial workers (8.8%), U.S. managers (7.0%), Norwegian restaurant workers (6.0%), Norwegian private sector employees (11.0%), Canadian employees (8.1%), and Japanese computer factory workers (males 13.0%, females 4.0%) [19–24]. These studies may, however, not be directly comparable as a result of application of different measures of alcohol consumption and different thresholds for risky drinking. Some [20, 22, 23] were solely based on number of consumed alcohol units during a specified time frame (e.g., number of units consumed during a typical drinking day, drinking frequency during the preceding year, and number of units each day during the preceding week), while others [19, 24] applied instruments with a broader scope (e.g., the Mortimer-Filkins test of problem drinking and the Kurihama Alcoholism Screening Test). Despite the use of different tools for conceptualisation and measurement of risky drinking, taken together these studies do suggest that risky drinking is an existing phenomenon among employees that deserves greater attention, given the adverse consequences associated with harmful alcohol consumption.

Early identification and intervention may be beneficial in preventing the development of alcohol problems. Knowledge on associations between sociodemographic factors and risky drinking may aid in determining which groups of employees that may need and benefit the most from early identification and interventions targeting alcohol-related problems. Some studies have demonstrated associations between risky drinking and sociodemographic variables, generally suggesting that risky drinking is more prevalent among younger individuals and males [14, 16, 17, 23], and that individuals with higher education are more prone to risky drinking than individuals with lower education [16, 17]. Although findings are more inconsistent, some authors have demonstrated associations between living/marital status and risky drinking [11, 14, 23].

The majority of the adult population is employed and employees with a risky drinking pattern constitute a much larger group than heavy drinkers [25]. The workplace may therefore be an important arena for identification and implementation of interventions targeting risky drinking. It seems imperative to produce more knowledge on risky drinking in the working community, on the factors that characterise workers who are at particular risk of developing alcohol problems, and on intervention approaches that might be beneficial. Overall, research is rather scarce on risky drinking among employees and there is a general lack of recent studies. Updated knowledge is imperative, since drinking behaviour results from a complex set of dynamic and interacting antecedents [26], some of which are susceptible to changes over time. For instance, Mäkelä et al. [27] found, in a Finnish study, a fundamental cultural shift in alcohol consumption over time, particularly for women and people aged over 30 years, and Allamani et al. [28] emphasise a changing Western drinking pattern characterised by increased beer and spirits consumption in social settings during evenings and weekends. Moreover, research tends to be characterised by not utilising internationally validated alcohol screening instruments [20, 22, 23], by being limited to specific subgroups within the workforce (specific sectors, industries or workers versus managers) [19–24], or by applying validated screening instruments without explicitly investigating practical implications and intervention needs in accordance with international intervention guidelines [19–22, 24]. The present study adds to the existing literature by providing updated knowledge, based on a recent sample of employees not restricted to specific subgroups, by utilising an internationally validated alcohol screening instrument, and by explicitly exploring implications for intervention needs in the workforce in accordance with international guidelines.

The aims of the study were therefore to (a) explore the proportions of risky drinkers in a sample of Norwegian employees by utilising an internationally validated alcohol screening instrument, (b) investigate sociodemographic associations with risky drinking, and (c) examine implications for intervention needs, based on World Health Organization guidelines.

## Methods

### Design and setting

The present study is one of several studies in the Norwegian national WIRUS-project (Workplace Interventions preventing Risky Use of alcohol and Sick leave). Other results from the WIRUS-project are published elsewhere [29, 30]. This study was designed as a cross-sectional alcohol screening study among private (*n* = 5) and public companies (*n* = 9) in Norway, employing approximately 14.353 individuals.

Alcohol consumption in the general Norwegian population per person per year (7.7 l) is somewhat lower compared to the rest of Europe (10.9 l) and the United States (9.2 l) [2]. Alcohol is a legal substance in Norway. However, restrictive policies and regulations are implemented (e.g., alcohol sale monopoly, age limits, advertising ban and taxation on products containing alcohol) [31]. Alcohol is forbidden in the workplace and infringement may result in resignation [32].

### Data collection and sample

4432 employees (30.9%) responded on a web-based questionnaire designed to measure alcohol consumption as well as sociodemographic variables. 3571 employees, 32.6% males and 67.4% females, responded on all items (24.9%), and thus constitute the sample in the present study. Study sample and invited sample characteristics are presented in Table 1.

**Table 1:** Study sample and invited sample characteristics

Variable	Study sample % (n)	Invited sample % (n)	Difference Percentage points (*p-value*)
Gender			
Male	32.6 (1164)	34.2 (4908)	- 1.6 (ns)
Female	67.4 (2407)	65.8 (9445)	+ 1.6 (ns)
Age			
≤ 39	31.3 (1116)	35.5 (5102)	- 4.2 (< .001)
≥ 40	68.7 (2455)	64.5 (9251)	+ 4.2 (< .001)
Variable		Study sample % (n)	
Educational level	Primary/lower secondary	2.4	85
	Upper secondary	22.7	809
	University/college	75.0	2677
Living status	Living alone	13.9	496
	Living with others	86.1	3075
Marital status	Unmarried	43.5	1553
	Married	56.5	2018
Children	No	20.5	731
	Yes	79.5	2840
Children in household	No	43.1	1538
	Yes	56.9	2033
Work position	Worker^a^	81.7	2918
	Manager	18.3	653
Work division^b^	Transportation	1.8	63
	Manufacturing	5.3	191
	Public administration	75.5	2697
	Health care services	16.6	593
	Accommodation	0.8	27

^a^Category includes blue, white and pink collar workers; ^b^Classification based on the European Classification of Economic Activities [49]

Approximately seven out of ten employees were aged 40 or older and had completed a university or college education. Only 13.9% of employees lived alone, while nearly half (43.5%) of the sample was unmarried. Almost eight out of ten employees had children, while close to six out of ten had children living in their household. Approximately two out of ten employees were classified as managers, and the majority of employees were employed within public administration (75.5%) and health care services (16.6%). Information on gender and age distributions among all employees in the 14 companies (invited sample) was collected through the companies’ personnel records.

### Measures

Alcohol consumption was measured with the Norwegian version of the Alcohol Use Disorders Identification Test (AUDIT), developed by the WHO [8]. The AUDIT is a widely used tool for identifying risky drinking and consists of ten questions concerning recent alcohol use, alcohol dependence symptoms and alcohol-related problems, each item with a potential score range from 0 to 4. A total score of ≥8 indicates the presence of risky drinking, and studies have demonstrated that this cut off carries favorable sensitivity and acceptable specificity [8]. Even though some studies have indicated that different thresholds for risky drinking should be applied for different groups (e.g., for males and females), a score of ≥8 has generally been accepted as an optimal cut off for identifying risky drinking [8, 33]. The threshold between low-risk and risky drinking was set at ≥8 scores on the AUDIT, and risky drinking was categorised in three risk levels, based on total scores on the AUDIT, each with a recommended procedure for intervention. Individuals with moderate risk (AUDIT scores 8–15) should be given simple advice on how to reduce risky drinking, individuals with high risk (AUDIT scores 16–19) should be provided with brief counselling and consecutive monitoring, and individuals with dependence likely risk (AUDIT scores 20–40) should be referred to further diagnostic evaluation for alcohol dependence [8, 33].

The AUDIT has been referred to as the global gold standard of alcohol screening instruments [34]. It is designed for international use, it is developed on the basis of data from a multinational sample, and it has been validated across countries and populations, with estimates of internal consistency (Cronbach’s α) typically ranging from 0.59 to 0.97 [35], yielding a mean alpha coefficient of 0.80 [33]. In the present study, internal consistency for the ten AUDIT items was estimated to 0.72, with a mean inter-item correlation of 0.25. Obtained psychometric estimates for the AUDIT in the present study were deemed satisfactory and in line with findings from previous studies [33, 35].

Gender (male/female), living status (living alone/living with others), marital status (unmarried/married), children (having no children/having children), children in the household (having no children at home/having children at home) and work position (worker/manager) were coded as dichotomous categorical variables. Age was collapsed into categories (≤39/≥40) for application in chi square test for independence, and treated as a continuous variable in the logistic regression model. Educational level was collapsed into two categories (without/with higher education) for utilisation in chi square test for independence, and treated as a categorical variable with four levels (primary/lower secondary, upper secondary, university/college < 4 years, university/college > 4 years) in the logistic regression model.

### Analysis

Proportions of risky drinking were estimated by calculating the proportion of employees exceeding the cut off (≥8 scores) on the AUDIT, for the overall sample as well as cross-tabulated proportions for males, females and both genders according to age, educational level, living status, marital status, number of children, number of children living in the household and work position. Bivariately, a series of chi square tests for independence were applied in order to explore whether gender, age, educational level, living status, marital status, number of children, number of children living in the household and work position were significantly associated with risky drinking. Next, adjusted logistic regression was conducted to assess the influence of the sociodemographic variables on the likelihood that employees would report risky drinking. Implications for intervention needs and approaches were investigated by calculating the proportions of risky drinkers in risk levels according to sum scores on the AUDIT (moderate risk: scores 8–15; high risk: scores 16–19; dependence likely: scores 20–40), and evaluating the risk level distributions in accordance with World Health Organization intervention recommendations.

All statistical analyses were performed with IBM SPSS version 24. Significant results were defined as *p* < .05.

### Ethics

Respondents were informed about the study’s aim, assured confidentiality and that participation was voluntary. We further collected written informed consent. The study was approved by the Regional Committee for Medical and Health Research in Norway (REK) (approval no. 2014/647).

## Results

### Proportions of risky drinkers

3179 employees (89.0%) scored within the low-risk category, while 392 employees (11.0%) had an AUDIT score equal to or above the cut off. Cross-tabulated proportions of risky drinking for males, females and both genders according to age, educational level, living status, marital status, children, children in househould and work position are presented in Table 2. A higher percentage of males compared to females were identified as risky drinkers (18.1% versus 7.5%). For both genders, rates of risky drinking were higher among employees aged ≤39 (16.7%) versus employees aged ≥40 (8.4%), employees with primary or secondary education (12.9%) versus university/college education (10.3%), employees living alone (18.3%) versus living with others (9.8%), unmarried (15.6%) versus married employees (7.4%), employees without (22.6%) versus those with children (8.0%), employees without children in the household (14.6%) versus those with children living at home (8.2%), and employees characterised as workers (11.3%) versus managers (9.3%).

**Table 2:** Proportions of risky drinking (AUDIT ≥8) for males, females and both genders according to sociodemographics

	Males (%)	Females (%)	Both genders (%)
	18.1	7.5	11.0
Age			
≤ 39	26.5	12.4	16.7
≥ 40	14.7	5.2	8.4
Educational level			
Primary/secondary	21.3	8.6	12.9
University/college	17.0	7.2	10.3
Living status			
Living alone	31.4	12.4	18.3
Living with others	16.1	6.7	9.8
Marital status			
Unmarried	26.3	10.9	15.6
Married	12.4	4.8	7.4
Children			
No children	33.5	16.8	22.6
Children	13.8	5.2	8.0
Children in household			
No children in household	23.2	10.5	14.6
Children in household	14.3	5.3	8.2
Work position			
Worker	19.6	7.9	11.3
Manager	13.8	5.4	9.3

Risky drinking was found to be most widespread among males without children (33.5%), males living alone (31.4%), and males aged ≤39 (26.5%). Risky drinking was least widespread among married females (4.8%), females with children (5.2%) and females aged ≥40 (5.2%).

### Sociodemographic associations with risky drinking

A series of unadjusted chi square tests for independence demonstrated statistically significant bivariate associations between risky drinking and gender (χ^2^ (1, *n* = 3571) = 90.34, *p* < .001, *phi* = 0.16), age (χ^2^ (1, n = 3571) = 53.77, *p* < .001, *phi* = 0.12), educational level (χ^2^ (1, *n* = 3571) = 4.34, *p* < .05, *phi* = 0.04), living status (χ^2^ (1, *n* = 3571) = 32.01, *p* < .001, *phi* = 0.10), marital status (χ^2^ (1, n = 3571) = 61.33, *p* < .001, *phi* = 0.13), children (χ^2^ (1, n = 3571) = 126.44, *p* < .001, *phi* = 0.19), and children in household (χ^2^ (1, n = 3571) = 36.87, *p* < .001, *phi* = 0.10). Employees’ work position was not significantly associated with risky drinking (χ^2^ (1, n = 3571) = 2.19, *p* > .05, *phi* = .03).

The adjusted multivariate logistic regression model was statistically significant, χ^2^ (8, n = 3571) = 238.19, *p* < .001, indicating that the model was able to distinguish between employees who reported risky drinking and those who did not. The model explained between 6.5% (Cox and Snell *R*^2^) and 12.9% (Nagelkerke *R*^2^) of the variance in risky drinking, and correctly classified 88.8% of cases. As shown in Table 3, five independent variables made unique statistically significant contributions to the model. Gender displayed an odds ratio of 2.97 (*p* < .001), indicating that male employees were almost three times as likely as female employees to report risky drinking, adjusted for all other variables in the model. For each year of age less, the odds ratio for reporting risky drinking increased by a factor of 1.03 (*p* < .001), while for each decreasing unit of education, the odds of risky drinking increased by a factor of 1.17 (*p* < .05). With an odds ratio of 1.38 (*p* < .05), unmarried employees were more likely than married employees to be risky drinkers. Employees without children had a greater odds (1.62, *p* < .05) for risky drinking compared to employees with children.

**Table 3:** Associations between sociodemographic factors and risky drinking (AUDIT≥8). Multivariate logistic regression model

				95% CI for OR	
Variable	B	S.E.	OR	Lower	Upper
Gender (males are the ref.)	1.09	0.11	2.97***	2.37	3.71
Age	0.03	0.01	1.03***	1.02	1.04
Educational level	0.16	0.07	1.17*	1.03	1.34
Living status (living alone is the ref.)	0.17	0.17	1.18	0.85	1.63
Marital status (unmarried is the ref.)	0.32	0.14	1.38*	1.05	1.82
Children (no children is the ref.)	0.48	0.21	1.62*	1.08	2.43
Children in household (no children is the ref.)	0.30	0.18	1.34	0.95	1.90
Work position (worker is the ref.)	0.06	0.16	1.06	0.78	1.45

*p < .05; ***p < .001

There were tendencies for employees living alone and not having children in the household to have greater odds for risky drinking, compared to employees living with others and with children in the household. These associations, however, did not reach statistical significance when adjusting for all other factors in the model. Employees’ working position demonstrated neither a bivariate or a multivariate association with risky drinking.

### Implications for intervention approaches

Employees’ risk level assessments for the overall sample as well as for identified at-risk groups (males without children, males living alone and males aged ≤39), categorised by AUDIT sum scores and intervention recommendations, are presented in Table 4. Of those employees identified as risky drinkers in the overall sample (11.0%), 94.6% scored within the moderate risk category (AUDIT sum scores 8–15), wherein simple advice is the recommended intervention. Only 4.1 and 1.3% of risky drinkers scored within high risk (AUDIT sum scores 16–19) and the dependence likely category (AUDIT sum scores 20–40), respectively, with corresponding intervention recommendations of brief counselling/consecutive monitoring and diagnostic evaluation for alcohol dependence. Similarly, within identified at-risk groups. The vast majority of risky drinkers scored within the range of moderate risk (males without children: 95.3%; males living alone: 98.0%; males aged ≤39: 93.3%).

**Table 4:** Employees’ risk level assessment, categorised by AUDIT sum scores and intervention recommendations

Overall sample					
Risk level	AUDIT sum	Recommended intervention^a^	N	% of overall sample	% of risky drinkers
Low	0–7	Alcohol education	3179	89.0	–
Moderate	8–15	Simple advice	371	10.4	94.6
High	16–19	Brief counselling and consecutive monitoring	16	0.4	4.1
Dependence likely	20–40	Diagnostic evaluation for alcohol dependence	5	0.2	1.3
Identified at-risk groups (% of risky drinkers with moderate, high and dependence risk)					
Group			Moderate (AUDIT 8–15)	High (AUDIT 16–19)	Dependence likely (AUDIT 20–40)
Males without children			95.3	3.5	1.2
Males living alone			98.0	0.0	2.0
Males aged ≤39			93.3	6.7	0.0

^a^See [8, 48]

## Discussion

The aims of the present study were to explore the proportions of risky drinkers in a sample of Norwegian employees by utilising an internationally validated alcohol screening instrument, to investigate sociodemographic associations with risky drinking, and to examine implications for intervention needs based on WHO guidelines. The following main findings will be discussed: (a) Overall, approximately one out of ten employees reported risky drinking, (b) risky drinking was found to be associated with and most common among males, younger employees, employees with low education, unmarried employees and employees without children, and (c) the majority of identified risky drinkers scored within the lowest defined risk level, i.e., with moderate risk that may be addressed by means of low-cost interventions.

Most comparable to our study, Halkjelsvik and Storvoll [17] did find, by also utilising an AUDIT threshold of ≥8, risky drinking estimates in the general Norwegian population (17.0%) that were markedly higher than those found in our sample of Norwegian employees. However, they did include students and unemployed in their sample, which may contribute to explaining their higher estimates, given that studies have found particularly high levels of alcohol consumption within these groups [14, 18, 36, 37]. In our study, women were also somewhat overrepresented, which could explain lower prevalence estimates of risky drinkers.

Still, the present study found estimates of risky drinking (11.0%) marginally higher than what has been found in several other studies of employees, with estimates ranging from 6.0 to 8.8% [19–21, 23]. Our estimates are in line with what was found by Nesvåg and Lie when they studied Norwegian private sector employees [22]. Their study is not directly comparable to ours, as they did not include public sector employees in their sample. Moreover, they measured risky drinking solely on the basis of number of consumed alcohol units. In general, differences in estimates across studies may be due to actual prevalence differences within populations, or be a result of different studies employing different measures of alcohol consumption and risky drinking thresholds.

In accordance with previous studies [14, 16, 17, 23], we found that risky drinking was associated with being male and young. Compared to female employees, male employees were almost three times as likely to report risky drinking, while each year of decreased age was associated with an increased odds of risky drinking. A consistent finding is that men drink more and heavier than women, and that larger proportions of females compared to males are abstainers [38]. Such universality could imply that endogenous gender differences may play a role, even though drinking patterns are probably heavily moderated by social and cultural factors.

Even though evidence on the relationship between age and alcohol consumption is somewhat inconclusive, cross-sectional studies have demonstrated lower consumption levels at older ages, and longitudinal studies have revealed decreased consumption and drinking prevalence with increasing age [39]. Also, heavy episodic drinking (binge drinking) has been found to be most common among young males [40], which may contribute to explain the association between being young, male and a risky drinker. Consistent with previous research [11, 14, 23], we found that unmarried employees and employees without children were more likely to be risky drinkers compared to those married and with children. It may well be that living with a spouse or partner and having children act as protective factors against high levels of alcohol consumption.

In line with similar studies [16, 17], the present study found an association between educational level and risky drinking. However, we found an association in the opposite direction of most studies, i.e., we found that employees with lower education were more vulnerable to risky drinking than employees with higher education. It is unclear which mechanisms underlie the relationship between educational level and risky drinking. Consistent with our findings, Crum et al. [41] revealed that high-school drop outs were significantly more likely to develop alcohol dependence than individuals with a college degree. It has been proposed that individuals with low socioeconomic status are less adherent to public health recommendations than those with higher socioeconomic status [42]. In a large sample drawn from a general Danish population, Shnohr et al. [43] found that individuals with low education were more frequently classified as heavy drinkers, compared to individuals with higher education. The association between educational attainment and risky drinking among employees should be subject to further research.

In line with Halkjelsvik and Moan [17], we found that the majority of risky drinkers (both within the overall sample and within identified at-risk groups) scored within the lowest risk category (moderate risk), where simple advice is the recommended intervention strategy. Studies have in general revealed higher estimates of alcohol consumption and risky drinking among primarily non-working populations, e.g., unemployed and students [14, 18, 36, 37], compared to working populations [19–24]. Alcohol consumption corresponding to higher risk levels may be largely incompatible with functioning in a workplace over time. Heavy drinkers may, to a large extent, have been excluded from the working community due to alcohol problems. This may contribute to explain why we found that the majority of risky drinkers among employees can be characterised by moderate risk of developing alcohol problems rather than being heavy drinkers.

### Methodological considerations

The present study has some limitations. Conducted within a cross-sectional design, we have identified associations between sociodemographic variables and risky drinking. It is, however, not possible to draw causal conclusions from these associations. Moreover, risky drinking may be influenced by a great variety of variables not measured and included in this study.

The present study was based on a relatively large sample (*n* = 3571) from the Norwegian working community. The final response rate, however, was low (24.9%). Comparisons between the study sample and the invited sample (see Table 1) did, however, reveal very small differences regarding gender and age distributions. Distribution of gender in the study sample was not significantly different from the true distribution in the invited sample. Distribution of age was significantly different (*p* < .001), with a 4.2 percentage point overrepresentation of employees aged 40 and older. Generalisations should therefore be done with caution. Low response rates may contribute to prevalence estimates biased by non-response, and non-response bias may be a greater threat to prevalence estimates than to associations between variables [44]. Although evidence is somewhat inconclusive, it has been proposed that heavy drinkers, males and individuals with low socioeconomic status tend to be overrepresented among non-responders in health surveys [41–46]. Hence, non-response bias may have contributed to an underestimation of risky drinking in the present study.

The study benefited from utilising the AUDIT as a measure of alcohol consumption, a widely validated tool [33, 35], designed for international use across gender, age and cultures [8]. Moreover, the AUDIT did demonstrate satisfactory psychometric properties in the present study. The AUDIT does, however, suffer from limitations as a result of being a self-reported measure, and in the present study we were not able to compare AUDIT scores with a more objective measure of alcohol consumption. Self-reported alcohol consumption has been found to be considerably lower than estimates of actual alcohol sales [47]. Individuals who responded on the AUDIT questions may have underreported their actual alcohol consumption, possibly contributing to an underestimation of risky drinking in the present study.

### Implications

Sociodemographic associations identified in this study do imply that in some groups, a much larger proportion, (up to one in three) may be particularly exposed to risky drinking, i.e., males, young employees, employees with low education, unmarried employees and those without children. However, identified sociodemographic correlates may not be conceived of as a check list that can inform employers about each employee’s level of alcohol risk. Knowledge of a set of significant correlates of risky drinking may, on the other hand, be directive in determining which and to what extent companies should make alcohol-preventive measures an overall priority, and for early identification purposes.

In the present study, more than nine out of ten risky drinkers scored within the lowest risk category, implying that low-cost interventions (such as simple advice) on an individual and/or collective level may be serviceable and sufficient for the majority of risky drinkers. On an individual level, such interventions may be administered by the occupational health services or primary health care services. A stepped-care approach [8, 48] may be utilised, i.e., employees are first managed by means of the lowest intervention level according to their AUDIT score and referred to the next level if they do not respond to the initial intervention. On a collective level, companies may benefit from developing and implementing specific guidelines regarding work-related alcohol use, as well as establishing alcohol prevention as an integrated part of the continuous work on health safety and environment in the workplace.

The present study carries some implications for future research. In the research literature, it is evident that a variety of measures of alcohol consumption and thresholds for risky drinking are employed and few studies have ulitised internationally validated instruments. Such diversity and lack of standardisation makes it difficult to compare studies and assess whether observed differences are due to actual variation within populations or differences in measurement and conceptualisation. Future research should attempt at establishing more consensus on how to measure and conceptualise risky drinking.

In this study we found that lower education was associated with risky drinking. This finding contradicts previous findings that generally suggests an association in the opposite direction. Hence, future research would benefit from engaging in a more thorough exploration of the association between educational level and risky drinking, e.g., by means of longitudinal studies and studies investigating possible moderators and mediators of this relationship.

## Conclusions

A considerable amount of employees (between one and three out of ten) were identified as potential risky drinkers. Being male, young, having low education, being unmarried and not having children seem to characterise employees at particular risk. However, as many as nine out of ten risky drinking employees scored within the lowest risk level. Potentially, low-cost workplace-based interventions may be a cost-effective measure to meet a major challenge that faces individual employees, employers as well as larger society.

## Abbreviations

AUDIT: The Alcohol Use Disorders Identification Test
CI: Confidence interval
OR: Odds ratio
REK: Regional Committees for Medical and Health Research in Norway
S.E.: Standard error
WHO: World Health Organization
WIRUS: Workplace Interventions preventing Risky Use of alcohol and Sick leave

## References

[1] Lim SS, Vos T, Flaxman AD, Danaei G, Shibuya K, Adair-Rohani H (2012). A comparative risk assessment of burden of disease and injury attributable to 67 risk factors and risk factor clusters in 21 regions, 1990–2010: a systematic analysis for the global burden of disease study 2010. Lancet.

[2] World Health Organization (2014). Global status report on alcohol and health, 2014.

[3] Frone MR (2006). Prevalence and distribution of alcohol use and impairment in the workplace: a US national survey. J Stud Alcohol.

[4] Schou L, Moan IS (2016). Alcohol use–sickness absence association and the moderating role of gender and socioeconomic status: a literature review. Drug Alcohol Rev.

[5] Kirkham HS, Clark BL, Bolas CA, Lewis GH, Jackson AS, Fisher D (2015). Which modifiable health risks are associated with changes in productivity costs?. Popul Health Manag.

[6] Mangione TW, Howland J, Amick B, Cote J, Lee M, Bell N (1999). Employee drinking practices and work performance. J Stud Alcohol.

[7] Schultz AB, Edington DW (2007). Employee health and presenteeism: a systematic review. J Occup Rehabil.

[8] Babor TF, Higgins-Biddle JC, Saunders JB, Monteiro MG (2001). AUDIT: The alcohol use disorders identification test: guidelines for use in primary health care.

[9] Taylor SE (2009). Health psychology.

[10] Skogen JC, Sagvaag H, Sikveland B (2014). Alkohol og permanent arbeidsuførhet. Høyt forbruk av alkohol eller alkoholproblemer - hva er forskjellen? [alcohol and permanent work disability. High consumption of alcohol or alcohol problems - what is the difference?]. Alkohol + arbeidsliv = sant? En vitenskapelig antologi [alcohol + employment = true? A scientific anthology].

[11] Fleming MF, Manwell LB, Barry KL, Johnson K (1998). At-risk drinking in an HMO primary care sample: prevalence and health policy implications. Am J Public Health.

[12] Levola J, Aalto M (2015). Screening for at-risk drinking in a population reporting symptoms of depression: a validation of the AUDIT, AUDIT-C, and AUDIT-3. Alcohol Clin Exp Res.

[13] Rumpf H-J, Hapke U, Meyer C, John U (2002). Screening for alcohol use disorders and at-risk drinking in the general population: psychometric performance of three questionnaires. Alcohol Alcoholism.

[14] Shah AA, Bazargan-Hejazi S, Lindstrom RW, Wolf KE (2009). Prevalence of at-risk drinking among a national sample of medical students. Subst Abus.

[15] Waern M, Marlow T, Morin J, Östling S, Skoog I (2013). Secular changes in at-risk drinking in Sweden: birth cohort comparisons in 75-year-old men and women 1976–2006. Age Ageing.

[16] Yan T, Xu H, Ettner SL, Barnes AJ, Moore AA (2014). At-risk drinking and outpatient healthcare expenditures in older adults. J Am Geriatr Soc.

[17] Halkjelsvik T, Storvoll EE (2014). Andel av befolkningen i Norge med et risikofylt alkoholkonsum målt gjennom alcohol use disorders identification test (AUDIT) [proportion of the population in Norway with an at-risk alcohol consumption measured by alcohol use disorders identification test (AUDIT)]. Nord Stud Alcohol Dr.

[18] Myrtveit SM, Askeland KG, Knudsen AK, Knapstad M, Olsen R, Nedregård T (2016). Risky drinking among Norwegian students: associations with participation in the introductory week, academic performance and alcohol-related attitudes. Nord Stud Alcohol Dr..

[19] Webb GR, Redman S, Hennrikus D, Rostas JA, Sanson-Fisher RW (1990). The prevalence and sociodemographic correlates of high-risk and problem drinking at an industrial worksite. Addiction.

[20] Howland J, Mangione TW, Kuhlthau K, Bell N, Heeren T, Lee M (1996). Work-site variation in managerial drinking. Addiction.

[21] Kjaerheim K, Mykletun R, Aasland OG, Haldorsen T, Andersen A (1995). Heavy drinking in the restaurant business: the role of social modelling and structural factors of the work-place. Addiction.

[22] Nesvåg S, Lie T (2004). Rusmiddelbruk blant ansatte i norsk privat arbeidsliv arbeidsliv [Drug use among employees in Norwegian private sector]. Nordisk Alkohol- og Narkotikatidsskrift.

[23] Marchand A, Parent-Lamarche A, Blanc M-È (2011). Work and high-risk alcohol consumption in the Canadian workforce. Int J Env Res Pub He.

[24] Kawakami N, Harantani T, Hemmi T, Araki S (1992). Prevalence and demographic correlates of alcohol-related problems in Japanese employees. Soc Psych Psych Epid.

[25] Ames GM, Bennett JB (2011). Prevention interventions of alcohol problems in the workplace: a review and guiding framework. Alc Res Health.

[26] Frone MR (2003). Predictors of overall and on-the-job substance use among young workers. J Occup Health Psych.

[27] Mäkelä P, Tigerstedt C, Mustonen H (2012). The Finnish drinking culture: change and continuity in the past 40 years. Drug Alcohol Rev.

[28] Allamani A, Beccaria F, Voller F (2010). The puzzle of Italian drinking. Trends in alcohol consumption, harms and policy: Italy 1990–2010. Nord Stud Alcohol Dr.

[29] Nordaune K, Skarpaas LS, Sagvaag H, Haveraaen L, Rimstad S, Kinn LG, et al. Who initiates and organises situations for work-related alcohol use? The WIRUS culture study. Scand J Public Healt. 2017;45(8):749–756. 10.1177/1403494817704109 28666393

[30] Aas RW, Haveraaen L, Sagvaag H, Thørrisen MM. The influence of alcohol consumption on sickness presenteeism and impaired daily activities. The WIRUS screening study. PLoS One. 2017;12(10):e0186503. 10.1371/journal.pone.0186503 PMC5645115 29040323

[31] Norwegian Institute of Public Health (2016). Rusmidler i Norge 2016 [Drugs in Norway 2016].

[32] Sagvaag H, Sikveland B (2014). Alkohol + arbeidsliv = sant? En vitenskapelig antologi [Alcohol + employment = true? A scientific anthology].

[33] de Meneses-Gaya C, Zuardi AW, Loureiro SR, Crippa JAS (2009). Alcohol use disorders identification test (AUDIT): an updated systematic review of psychometric properties. Psychol Neurosci.

[34] Centers for Disease Control and Prevention (2014). Planning and implementing screening and brief intervention for risky alcohol use: a step-by-step guide for primary care practices.

[35] Reinert DF, Allen JP (2007). The alcohol use disorders identification test: an update of research findings. Alcohol Clin Exp Res.

[36] Janlert U, Hammarström A (1992). Alcohol consumption among unemployed youths: results from a prospective study. Addiction.

[37] Reine I, Novo M, Hammarström A (2013). Unemployment and ill health–a gender analysis: results from a 14-year follow-up of the northern Swedish cohort. Public Health.

[38] Wilsnack RW, Wilsnack SC, Kristjanson AF, Vogeltanz-Holm ND, Gmel G (2009). Gender and alcohol consumption: patterns from the multinational GENACIS project. Addiction.

[39] Eigenbrodt ML, Mosley TH, Hutchinson RG, Watson RL, Chambless LE, Szklo M (2001). Alcohol consumption with age: a cross-sectional and longitudinal study of the atherosclerosis risk in communities (ARIC) study, 1987–1995. Am J Epidemiol.

[40] Andersson A, Mårdby A-C, Holmgren K, Hensing G (2014). Associations between leisure activities and binge drinking in adults: findings from a Swedish newly sick-listed sample. Work.

[41] Crum RM, Helzer JE, Anthony JC (1993). Level of education and alcohol abuse and dependence in adulthood: a further inquiry. Am J Public Health.

[42] Lynch JW, Kaplan GA, Salonen JT (1997). Why do poor people behave poorly? Variation in adult health behaviours and psychosocial characteristics by stages of the socioeconomic lifecourse. Soc Sci Med.

[43] Schnohr C, Højbjerre L, Riegels M, Ledet L, Larsen T, Schultz-Larsen K (2004). Does educational level influence the effects of smoking, alcohol, physical activity, and obesity on mortality? A prospective population study. Scand J Soc Med.

[44] Knudsen AK, Hotopf M, Skogen JC, Øverland S, Mykletun A (2010). The health status of nonparticipants in a population-based health study: the Hordaland health study. Am J Epidemiol.

[45] Boniface S, Scholes S, Shelton N, Connor J (2017). Assessment of non-response bias in estimates of alcohol consumption: applying the continuum of resistance model in a general population survey in England. PLoS One.

[46] Korkeila K, Suominen S, Ahvenainen J, Ojanlatva A, Rautava P, Helenius H (2001). Non-response and related factors in a nation-wide health survey. Eur J Epidemiol.

[47] Boniface S, Kneale J, Shelton N (2014). Drinking pattern is more strongly associated with under-reporting of alcohol consumption than socio-demographic factors: evidence from a mixed-methods study. BMC Public Health.

[48] Babor TF, Higgins-Biddle JC (2001). Brief intervention for hazardous and harmful drinking: a manual for use in primary care.

[49] Eurostat (2008). NACE rev. 2. Statistical classification of economic activities in the European community.

